# Phase I study of chimeric antigen receptor modified T cells in treating HER2-positive advanced biliary tract cancers and pancreatic cancers

**DOI:** 10.1007/s13238-017-0440-4

**Published:** 2017-07-14

**Authors:** Kaichao Feng, Yang Liu, Yelei Guo, Jingdan Qiu, Zhiqiang Wu, Hanren Dai, Qingming Yang, Yao Wang, Weidong Han

**Affiliations:** 1 Department of Bio-therapeutic, Chinese PLA General Hospital, Beijing, 100853 China; 2 Department of Geriatric Hematology, Chinese PLA General Hospital, Beijing, 100853 China; 3 Department of Molecular & Immunology, Chinese PLA General Hospital, Beijing, 100853 China

**Keywords:** HER2, CART, biliary tract cancers, pancreatic cancers, clinical trial

## Abstract

This phase I clinical trial (NCT01935843) is to evaluate the safety, feasibility, and activity of chimeric antigen receptor-engineered T cell (CART) immunotherapy targeting human epidermal growth factor receptor 2 (HER2) in patients with advanced biliary tract cancers (BTCs) and pancreatic cancers (PCs). Eligible patients with HER2-positive (>50%) BTCs and PCs were enrolled in the trial. Well cultured CART-HER2 cells were infused following the conditioning treatment composed of nab-paclitaxel (100–200 mg/m^2^) and cyclophosphamide (15–35 mg/kg). CAR transgene copy number in the peripheral blood was serially measured to monitor the expansion and persistence of CART-HER2 cells *in vivo*. Eleven enrolled patients received 1 to 2-cycle CART-HER2 cell infusion (median CAR^+^ T cell 2.1 × 10^6^/kg). The conditioning treatment resulted in mild-to-moderate fatigue, nausea/vomiting, myalgia/arthralgia, and lymphopenia. Except one grade-3 acute febrile syndrome and one abnormal elevation of transaminase (>9 ULN), adverse events related to the infusion of CART-HER2 cells were mild-to-moderate. Post-infusion toxicities included one case of reversible severe upper gastrointestinal hemorrhage which occurred in a patient with gastric antrum invaded by metastasis 11 days after the CART-HER2 cell infusion, and 2 cases of grade 1–2 delayed fever, accompanied by the release of C-reactive protein and interleukin-6. All patients were evaluable for assessment of clinical response, among which 1 obtained a 4.5-months partial response and 5 achieved stable disease. The median progression free survival was 4.8 months (range, 1.5–8.3 months). Finally, data from this study demonstrated the safety and feasibility of CART-HER2 immunotherapy, and showed encouraging signals of clinical activity.

## Introduction

Pancreatic cancers (PCs) and biliary tract cancers (BTCs), which are composed of cholangiocarcinoma (CCA) and gallbladder carcinoma (GBCA), are diseases with similar embryologic origin, biological behavior, and pathological features (Cardinale V et al., 2012; Marks and Yee, 2016), and a consequent similar poor prognosis (Torre LA, et al., 2015). The high mortality of PC and BTCs is due to lack of early diagnosis and effective systemic treatment. Surgery is the only curative treatment option, however, majority of the patients at the time of diagnosis are candidates with unresectable, locally advanced, or metastatic disease, resulting the overall survival (OS) less than 12 months or even shorter (Ryan et al., 2014; Chan and Berlin 2015). Although chemotherapy remains the primary strategy in managing unresectable, locally advanced, relapsed, and metastatic PCs and BTCs, few chemotherapeutic options could demonstrate their benefits in significantly prolonging patients’ progression free survival (PFS) and OS. Therefore, there is still huge unmet need for the development of novel therapeutic strategies in treating advanced BTCs and PCs.

Recently, chimeric antigen receptors (CARs) engineered T cells specifically targeting CD19 or CD20 antigen have consistently demonstrated high antitumor efficacy across a range of B-cell hematological malignancies (Dai H et al., 2015; Wang Y et al., 2014; Kochenderfer JN et al., 2015; Park JH et al., 2016; Maus and June 2016). Based on this success, there is now mounting interest around how to use CAR T cells for the treatment of solid malignancies. However, due to the reliance on cell surface protein recognition, the selection of a target protein that is highly specific tumor associated antigen (TAA) can be quite challenging (Abken H, 2015; Klebanoff CA et al, 2016).

Human epidermal growth factor receptor 2 (HER2) is a transmembrane glycoprotein that belongs to the family of epidermal growth factor receptor, mediating cell proliferation and differentiation in the developing embryo and in adult tissues (Cho HS et al., 2003). However, overexpression of HER2 plays a central role in tumorigenesis of numerous human cancers and is associated with more aggressive clinical behavior (Mendelsohn and Baselga, 2000). HER2 overexpression is observed in approximately 20%–70% of BTCs and 7%–58% of PCs (Ogo Y et al., 2006; Yan M et al., 2015; Zhang Z et al., 2010; Nam AR et al., 2016; Zhang Y et al., 2006), making it an ideal target protein for CAR-T cell therapy. Therefore, we designed this phase I clinical trial to evaluate the safety, feasibility, and activity of CART cell therapy in HER2-overexpressing advanced unresectable, relapsed/metastatic BTCs and PCs.

## Results

### CART-HER2 cell product assessment

**Figure 1:**
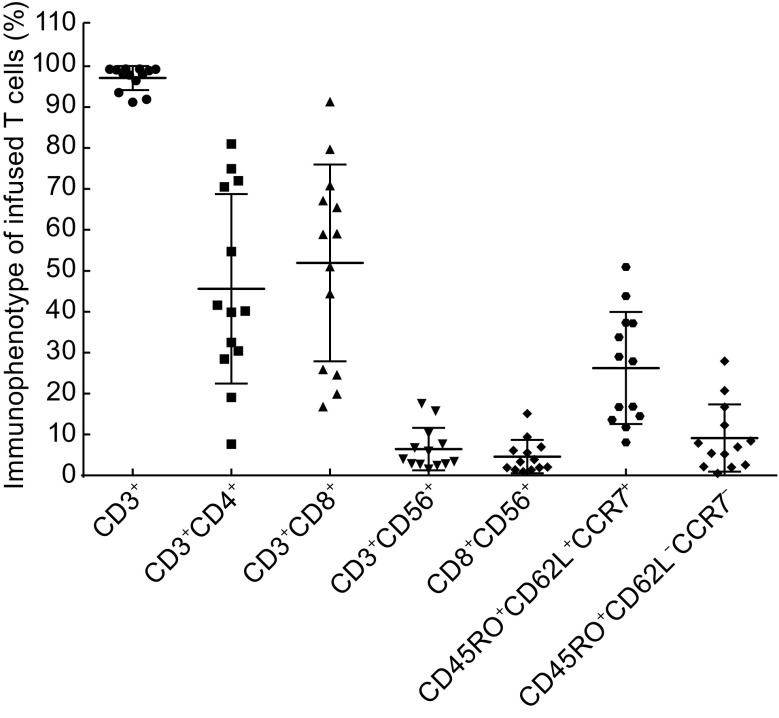
Phenotype of the cultured CART-HER2 cells

For all patients, the total CART-HER2 cells were harvested for infusion when reaching to a 20-fold expansion after a 10-day culture. Of the infused cells, 97.1% (median, range 91.5%–99.3%) were CD3^+^ cells principally composed of the CD8^+^ subset (median 51.9%, range 16.8%–91.3%), and 26.1% (median, range 8.1%–50.9%) were characterized with the central memory phenotype (CD45RO+/CD62L+/CCR7+) (Fig. 1). In addition, 9.9% (median, range 5.5%–11.4%) of the infused cells were CAR-HER2 positive.

### Patients’ general characteristics

From July 2015 to June 2016, a total of 11 patients with advanced unresectable, relapsed or metastatic BTCs and PCs were enrolled into the trial. General characteristics of each patient were presented detailedly in Table 1. The median age of enrolled patients was 61-years (range 50–75). Two out of 11 patients received the second cycle CART-HER2 immunotherapy. Patient No. 9 had to cancel the planned cyclophosphamide when a grade-3 thrombocytopenia occurred four days after the administration of nab-paclitaxel. Patient No. 11 canceled cyclophosphamide in his second cycle CART-HER2 cell therapy due to complicated upper respiratory infection occurring two days after the onset of conditioning chemotherapy. The median dose of CAR-HER2 positive T cells in each cycle was 2.1 × 10^6^/kg (range 1.4–3.8 × 10^6^/kg).

**Table 1:** Patients’ clinical characteristics

Patient No.	Sex	Age (years)	Diagnosis	Status at enrollment	Cycles of CART therapy	Conditioning regimens nab-P (mg/m^2^), CTX(mg/kg)	CAR-positive T cells in each cycle (×10^6^/kg)	Best response	Progress free survival (months)
1	Male	62	pCCA	Relapsed/Metastatic	1	nab-P 194.8. CTX 29.4	3.8	PR	4.5
2	Male	61	iCCA	Relapsed/Metastatic	1	nab-P 171.4. CTX 11.9	2.9	PD	
3	Male	59	iCCA	Relapsed/Metastatic	1	nab-P 225.7. CTX 8.0	2.9	PD	
4	Female	53	pCCA	Relapsed/Metastatic	2	nab-P 135.1. CTX 26.9 nab-P 135.1. CTX 23.1	1.9^(1st)^ 3.6^(2nd)^	SD	5.0
5	Male	53	iCCA	Relapsed/Metastatic	1	nab-P 125.0. CTX 14.3	1.6	PD	
6	Male	62	pCCA	Unresectable	1	nab-P 176.5. CTX 25.0	2.0	PD	
7	Male	75	GBCA	Unresectable	1	nab-P 119.1. CTX 26.7	1.4	PD	
8	Female	56	iCCA	Metastatic	1	nab-P 189.9. CTX 27.6	2.9	SD	1.5
9	Male	61	pCCA	Relapsed/Metastatic	1	nab-P 187.5.^★^	1.5	SD	2.0
10	Male	50	PC	Metastatic	1	nab-P 145.6. CTX 22.2	2.1	SD	5.3
11	Male	74	PC	Relapsed/Metastatic	2	nab-P 116.3. CTX 20.0 nab-P 115.6.^☆^	1.9^(1st)^ 3.4^(2nd)^	SD	8.3

1st: the first cycle of CART-HER2 treatment; 2nd: the second cycle of CART-HER2 treatment

*dCCA* distal cholangiocarcinoma; *iCCA* intrahepatic cholangiocarcinoma; *pCCA* perihilar cholangiocarcinoma; *GBCA* gallbladder carcinoma; *PC* pancreatic carcinoma; *nab-P* nab-paclitaxel; *CTX* cyclophosphamide; *PR* partial response; *SD* stable disease; *PD* progressive disease

^★^CTX was canceled for a grade-3 decrease of platelet. ^☆^CTX was canceled for complicated upper respiratory infection

### Toxicities

**Figure 2:**
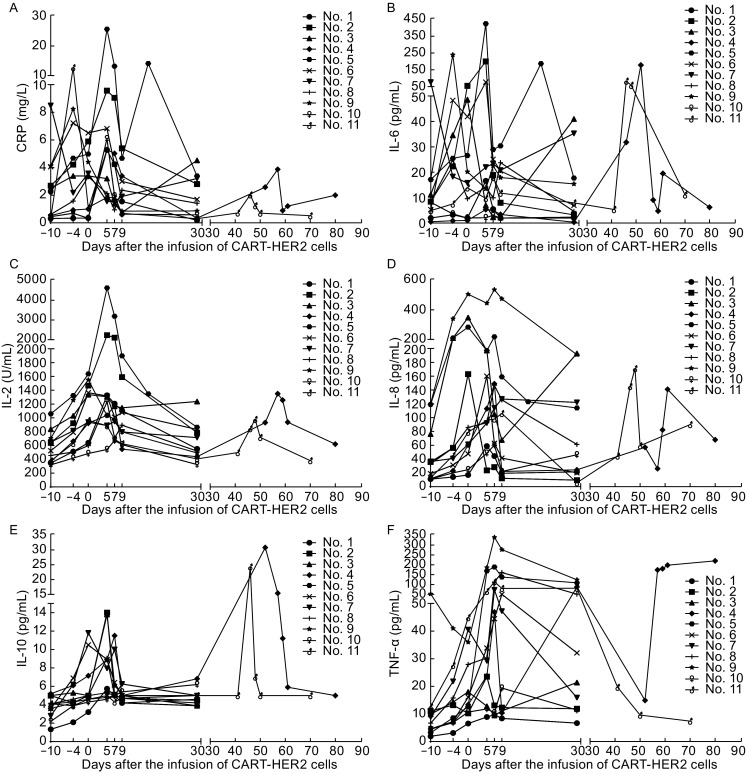
Release of CRP and cytokines at scheduled time points and occasional time points from patients’ peripheral blood since the enrollment of CART-HER2 cell therapy. (A) Change of CRP during the CART-HER2 cell therapy. (B) The level of IL-6 release monitored according to the study flowchart. (C) Release of interleukin-2 (IL-2). (D) Fluctuation of interleukin-8 (IL-8) following the treatment of CART-HER2 cell infusion. (E) Interleukin-10 (IL-10) detected after the CART-HER2 cell treatment. (F) Release of tumor necrosis factor-α (TNF-α)

Adverse events (AEs) that occurred in the process of CART-HER2 immunotherapy were categorized according to the Common Terminology Criteria for Adverse Events Version 4.0 (CTCAE 4.0) and summarized in 3 separate sections according to the study flowchart: conditioning chemotherapy-related toxicities, CART-HER2 cell infusion-related toxicities, and post-infusion toxicities (Table 2). AEs occurring during the period of conditioning chemotherapy included mild-to-moderate nausea/vomiting (72.7%), fatigue (63.6%), and myalgia/arthralgia (45.5%). Lymphopenia was another common toxicity which occurred in 81.8% patients, among which 54.5% experienced a grade 3–4 decrease of lymphocytes. Except one case of grade-3 acute fever/chill and one case of abnormal transaminase elevation (>9 ULN), AEs related to the infusion of CART cells were mild or moderate, among which acute febrile syndrome was the most frequent AE. Mild skin pruritus and upper gastrointestinal hemorrhage occurred in two patients respectively during the infusion of CART-HER2 cells and disappeared immediately when the CART-HER2 cell therapy was completed. Post-infusion toxicities included one case of reversible severe upper gastrointestinal hemorrhage which occurred in patient No. 5 with gastric antrum invaded by metastasis 11 days after the CART-HER2 cell infusion and 2 cases of delayed fever which occurred 2 days and 4 days respectively after the infusion of CART-HER2 cells, accompanied by the release of C-reactive protein (CRP) and cytokines (Fig. 2). 1.5-fold to 18.6-fold increase of CRP and interleukin-6 (IL-6) following the administration of CART-HER2 cells infusion were observed in 6/11 and 10/11 patients respectively (Fig. 2A and 2B), however, there was not severe cytokine release symptom (CRS) occurred in this trial. All toxicities associated with the CART-HER2 immunotherapy were reversible, and there was no treatment-related death.

**Table 2:** Adverse events related to CART-HER2 therapy

AEs	Conditioning section				Infusion section				Post-infusion section			
	Any grade		Grade 3–4		Any grade		Grade 3–4		Any grade		Grade 3–4	
	N	%	N	%	N	%	N	%	N	%	N	%
Oral mucositis	2	18.2										
Gastrointestinal hemorrhage					1	9.1			1	9.1	1	9.1
Skin pruritus	1	9.1			1	9.1						
Anemia	3	27.3			3	27.3						
Lymphopenia	9	81.8	6	54.5	3	27.3						
Thrombocytopenia	2	18.2										
Acute fever/Chill	2	18.2			11	100	1	9.1				
Delayed fever/Chill									2	18.2		
Fatigue	7	63.6			4	36.4						
Transaminase elevation					2	18.2	1	9.1				
Diarrhea					1	9.1						
Nausea/Vomiting	8	72.7			1	9.1						
Myalgia/Arthralgia	5	45.5

### Clinical response

**Figure 3:**
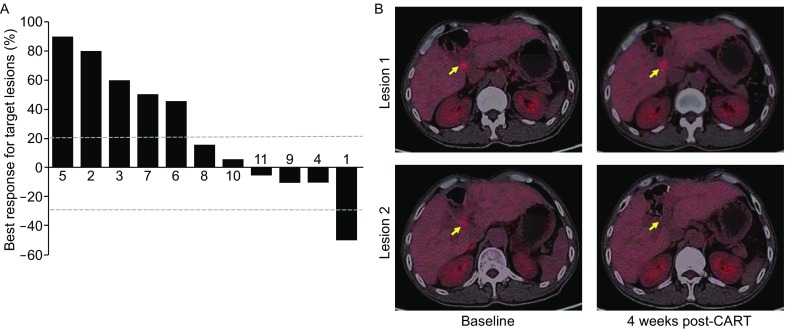
Clinical response. (A) Best overall response from baseline in the sum of the longest diameters of target lesions as assessed per RECIST 1.1 in patients who had at least one post-baseline tumor assessment. (B) Patient No. 1, a 62-year-old male with poorly differentiated perihilar CCA accompanied by Her2 protein overexpressed in >90% tumor cells, whose PET-CT showed 2 metastatic lesions in his hepatic hilum (yellow arrows) obtained a PR 4 weeks after one cycle of CART-HER2 therapy, assessed by PET-CT showing the disappearance of lesion 2

All enrolled patients received at least one post-baseline tumor assessment per Response Evaluation Criteria in Solid Tumors 1.1 (RECIST 1.1) and obtained 1 partial response (PR) and 5 stable disease (SD) (Fig. 3A). The median PFS was 4.8 months (range 1.5–8.3 months) (Table 1). Patient No. 1, a 62-year-old male with poorly differentiated perihilar CCA accompanied by Her2 protein overexpressed in >90% tumor cells, was enrolled into this study when 2 metastatic lesions in his hepatic hilum were detected by positron emission tomography-computed tomography (PET-CT) 12 months after the radical surgery. He obtained a PR assessed by PET-CT four weeks after one cycle of CART-HER2 therapy, showing the disappearance of one metastatic lesion and a decrease of the other lesion’s standardized uptake value (SUV) from 6.5 to 4.7 (Fig. 3B). His PFS maintained for 4.5 months.

### Expansion and persistence of CART-HER2 cells *in vivo*

**Figure 4:**
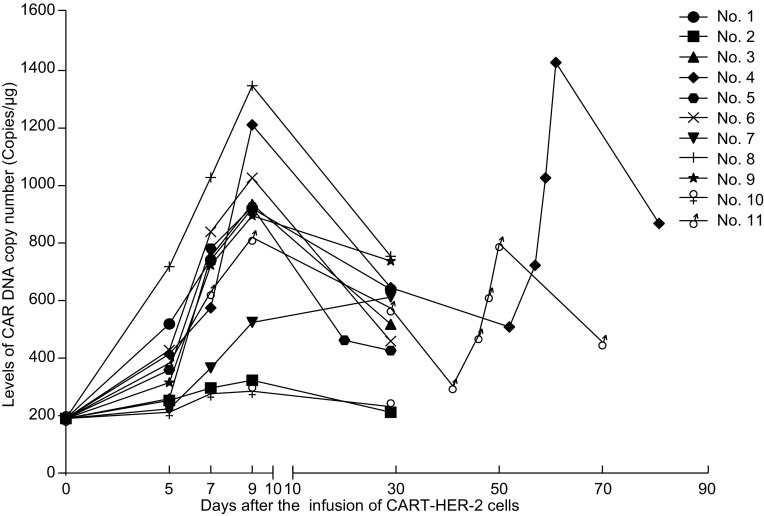
Change of CAR transgene copy number level in the peripheral blood following the infusion of CART-HER2 cells. Rapid elevation of CAR transgene copy numbers (>2.5-fold of the baseline value) was observed in 9/11 patients, meanwhile, 9/11 patients’ serum CAR transgene copy numbers were still above 2-fold of the baseline level. The CAR transgene copy number after the second cycle infusion of CART-HER2 cells could reach to a similar peak of the first cycle

The transgene copy number of CAR genomic DNA in the peripheral blood was detected at scheduled time points to monitor the expansion and persistence of CART-HER2 cells *in vivo*. Rapid elevation of CAR transgene copy numbers (>2.5-fold of the baseline value) was observed in 9/11 patients following the infusion of CART-HER2 cells, indicating robust expansion of CART cells *in vivo* (Fig. 4). Except 2 patients whose CAR transgene copy numbers declined to the baseline value within 1 month, 9/11 patients’ serum CAR transgene copy numbers were still above 2-fold of the baseline level at the first evaluation timepoint, showing that CART-HER2 cells could persist effectively *in vivo*. The CAR transgene copy number of patient No. 4 and patient No. 11 after the second cycle infusion of CART-HER2 cells reclimbed to the peak that were similar with that of the first cycle. Although patient No. 5 developed severe upper gastrointestinal hemorrhage 11 days after the CART-HER2 cell infusion accompanied by the elevation of CRP and IL-6, his CAR transgene copy number in the peripheral blood did not present synchronous elevation.

## Discussion

Targeting HER2 with CAR-redirected T cells is an attractive strategy to expand HER2 targeted immunotherapies to malignancies that are HER2 antigen positive but are insensitive to HER2 antibodies because they are not HER2 gene amplified (Ahmed N et al., 2015; Ahmed N et al., 2010). However, since Richard A Morgan first reported a lethal case in which a patient with colon cancer metastatic to the lungs and liver was treated with a dose of 10^10^ CART-HER2 cells, resulting in uncontrollable acute respiratory distress and subsequent death (Morgan RA et al., 2010), the investigation of CART cell immunotherapy targeting overexpressed HER2 antigen had to be suspended for the safety concern until Nabil Ahmed proved that HER2-specific CART cells were well tolerated with no dose-limiting toxicity in 19 patients with HER2-positive sarcoma (Ahmed N et al., 2015).

Different from the nonmyeloablative conditioning regimen adopted by Richard A Morgan and monotherapy of CART-HER2 cell infusion by Nabil Ahmed, we adopted a new conditioning regimen consisted of nab-paclitaxel and cyclophosphamide before the infusion of CART-HER2 cells in HER2 overexpressing advanced unresectable, relapsed/metastatic BTCs and PCs in this study for nab-paclitaxel was reported that it could deplete the desmoplastic stroma and increase vascularization in mice xenografts derived from PC patients (Von Hoff DD et al., 2011) and speculated to allow the infused CART cells to reach to the tumor tissues more efficiently. However, we lack biopsied tissues from tumor lesions after the CART-HER2 cell therapy to prove the efficiency of nab-paclitaxel in promoting the infiltration of CART cells, which will be implemented in the future study. In the assessment of safety, our toxicities profiles showed that the addition of nab-paclitaxel to cyclophosphamide did not bring severe toxicities to patients participating this trial.

The safety of CART-HER2 cells is the primary concern of our study. In this study, the infusion of CART-HER2 cells was safe without significant adverse events, one possible important reason is the dose-escalation infusion strategy in which well-cultured CART cells were harvested partially per day for infusion in 3–5 successive days instead of one-off infusion of the total CART cells, which may facilitate to avoid severe acute allergy or other CART cell infusion-related toxicities that may impair the function of pulmonary, cardiac, and other organs. However, two cases of upper gastrointestinal hemorrhage which occurred in the period of CART cell infusion and 11 days after the infusion of CART cells respectively warned us the on-target off-tumor threat of HER2-targeting CART cells because of the expression of HER2 antigen on gastrointestinal mucosa though with a lower density when compared with tumor tissues (Hynes and Lane, 2005).

Our preclinical study agreed with two other studies in which CAR-expressing T cells exhibited robust antitumor activity in targeting HER2 positive xenogeneic mouse tumor models (Liu X et al., 2015; Nakazawa Y et al., 2011). However, only 1 partial response was observed in this clinical study when assessed with the conventional RECIST 1.1 criteria. Meanwhile, five patients obtained a SD status after the CART-HER2 cell therapy, among which 3 patients’ PFS were longer than 5 months. If considering the highly aggressive biological behavior and pathological features of advanced BTCs and PCs, radiologic SD with long PFS may indicate the clinical benefit of CART immunotherapy. As others have reported, conventional RECIST criteria may underestimate the antitumor effects of immunotherapies (Axel Hoos et al., 2010). Immune-related response criteria may be a better evaluation tool to reflect the activity of immune therapy in solid tumors (Wolchok JD et al., 2009).

In hematological malignancies, robust *in vivo* expansion and persistence of CAR T cells is a critical determinant of therapeutic efficacy (Porter DL et al., 2015). However, efficient expansion and persistence of CART cells *in vivo* is still a major obstacle for solid tumors, which in turn limits the antitumor activity of CART cells (Beatty and O’Hara, 2016). In this study, we observed not only the post-infusion expansion of CART cells *in vivo*, but also persistence of CART-HER2 cells in the peripheral blood. The efficient expansion and persistence of CART cell may in part be attributable to the conditioning chemotherapy, in which nab-paclitaxel may promote HER2 antigen presentation by depleting tumor stroma and cyclophosphamide plays a key role in depleting regulatory T cells (Tregs), myeloid-derived suppressor cell (MDSC) as well as other inhibitory cells, and creating an appropriate “lymphoid space” that is devoid of regulatory mechanisms (Dudley ME et al., 2002). Nevertheless, we failed to obtain a longer persistence of CART-HER2 cells at therapeutic level *in vivo*, requiring better understanding of the biology determining the persistence of CART cells and corresponding strategies that may improve the expansion and persistence of CART cells in solid malignancies.

In summary, the results from our clinical trial show the safety, feasibility of nab-paclitaxel and cyclophosphamide combination conditioning treatment-based CART-HER2 cell therapy and encouraging signals of clinical activity. However, there are still plenty of known and unknown obstacles that limit the application of CART therapy in the fight against solid tumors (Johnson and June 2017). Along with the solution of these obstacles, CART cell immunotherapy will reveal its greatest potential of antitumor activity.

## Patients and methods

### Study design

This study, registered at www.clinicaltrials.gov as NCT 01935843 and approved by the ethics committee of the Chinese PLA General Hospital, was designed to assess the safety, feasibility, and efficacy of CAR-T cell therapy in HER2-positive advanced unresectable, relapsed/metastatic BTCs and PCs. The primary objective of this study was safety. The secondary objectives were overall response rate (ORR), PFS. The exploratory objectives included the expansion and persistence of CAR^+^ T cells *in vivo* and biomarkers that could possibly reflect the antitumor activity of CART-HER2 cells. All enrolled patients provided written informed consent in accordance with the Declaration of Helsinki. No commercial sponsor was involved in the study.

### Inclusion criteria

Patients with advanced unresectable, relapsed/metastatic BTCs and PCs must meet the criterion of HER2 protein overexpression, that is >50% tumor cells expressing HER2 protein confirmed by two senior pathologists using the Herceptest^TM^ (Dako) criteria. Other inclusion criteria included that patients had an Eastern Cooperative Oncology Group (ECOG) performance status of 0–1, at least one measurable target lesion, adequate cardiac and pulmonary function, adequate bone marrow reserve, and hepatic and renal functions as follows: absolute neutrophil count ≥1500/mm^3^, platelet count ≥100,000/mm^3^, hemoglobin ≥10 g/dL, ALT/AST < 2.5× ULN, total bilirubin < 1.5× ULN, and serum creatinine < 1.5× ULN. All enrolled candidates were ages 18 to 80 years.

### Exclusion criteria

Patients were excluded if their life expectancy was shorter than 3 months, or they had uncontrolled hypertension (> 160/100 mmHg), unstable coronary diseases, severe liver and kidney dysfunction, any types of primary immunodeficiency, active virus infections such as hepatitis and human immunodeficiency virus (HIV), or pulmonary function abnormalities as follows: forced expiratory volume (FEV) <30% predicted, diffusing capacity of lung for carbon monoxide (DLCO) <30% predicted (post-bronchodilator), oxygen saturation <90% on room air. Patients who were undergoing pregnancy or lactation or other clinical trials were excluded.

### Constrcution, generation, expansion, and cytotoxicity of CART-HER2 cells *in vitro*

The DNA sequence of anti-HER2 scFv-CD137-CD3ζ CAR, which contained anti-Her-2 scFv, human CD8a hinge, CD137, and CD3ζ signaling domains, was constructed based on our previously published CAR sequence (Dai H et al., 2015). The CAR.HER2-CD137ζ-GFP vector was constructed to verify the transduction efficiency by using a FACSCalibur flow cytometry (BD Biosciences, San Jose, CA, USA). The generation and expansion of CART cells was performed according to our published protocol (Wang et al., 2013). In the preclinical study, we identified the characterization of CART-HER2 cells, cell proliferation capacity and antitumor activity *in vitro*, the detailed data of which was reported by Dr. Song Yanjing (Song Y et al., 2017), demonstrating that CART-HER2 cells possess effective and persistent antitumor activity against multiple HER2 positive adenocarcinoma cell lines and xenografts in mice by specifically targeting HER2 protein.

### Anti-HER2 CART treatment

**Figure 5:**
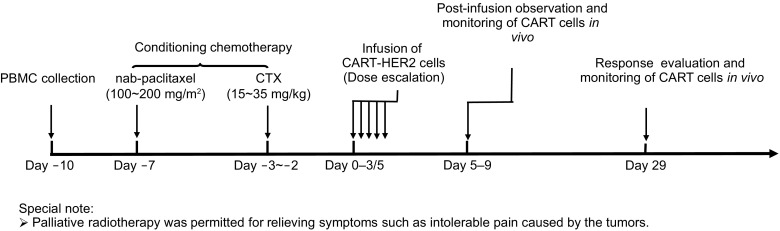
Study flowchart of CART-HER2 therapy. PBMC: peripheral blood mononuclear cell. CTX: cyclophosphamide

Each enrolled patient was required to provide 80–100 mL peripheral blood, from which autologous peripheral blood mononuclear cells (PBMCs) were purified for producing CART cells. Subsequently, all enrolled patients were administered with the conditioning chemotherapy composed of nab-paclitaxel (100–200 mg/m^2^ at d-7) and cyclophosphamide (15–35 mg/kg at d-3 to d-2). CART-HER2 cells were infused at d-0 in a manner of dose escalation over the following 3~5 days. Details of the protocol were showed in the study flow chart (Fig. 5). Samples of peripheral blood were collected for analyzing CAR transgene copy number and levels of cytokines such as IL-2, IL-6, IL-8, IL-10, interferon-gamma (IFN-gamma), tumor necrosis factor- alpha (TNF-alpha), vascular endothelial growth factor (VEGF), and Granzyme B at scheduled time points and occasional time points in which unexplained fever/chill or CRS or any other events that may be correlated to CART cells occurred. AEs were graded based upon the CTCAE 4.0. Clinical response was evaluated using contrast-enhanced computed tomography (CT), magnetic resonance imaging (MRI), positron emission tomography-computed tomography (PET-CT) according to the RECIST 1.1. Palliative radiotherapy was allowed for relieving the tumor-associated pain or other symptoms.

### Statistics

GraphPad Prism version 6.0 for Windows was applied to execute the statistical analysis. The outcomes were shown as means ± standard deviations (SDs). Descriptive statistics were used to summarize the data in multiplex analyses. Two-way analysis of variance was used to analyze the statistical difference between groups in all experiments. A *P* value less than 0.05 was considered statistically significant.
